# Supplementary material for: In-phase millennial-scale glacier changes in the tropics and North Atlantic regions during the Holocene
Source: Nat Commun. 2022 Mar 17;13:1419. doi: 10.1038/s41467-022-28939-9 (PMC8930989; doi:10.1038/s41467-022-28939-9)
Supplement: Supplementary file 1 — Supplementary Information [file 41467_2022_28939_MOESM1_ESM.pdf]

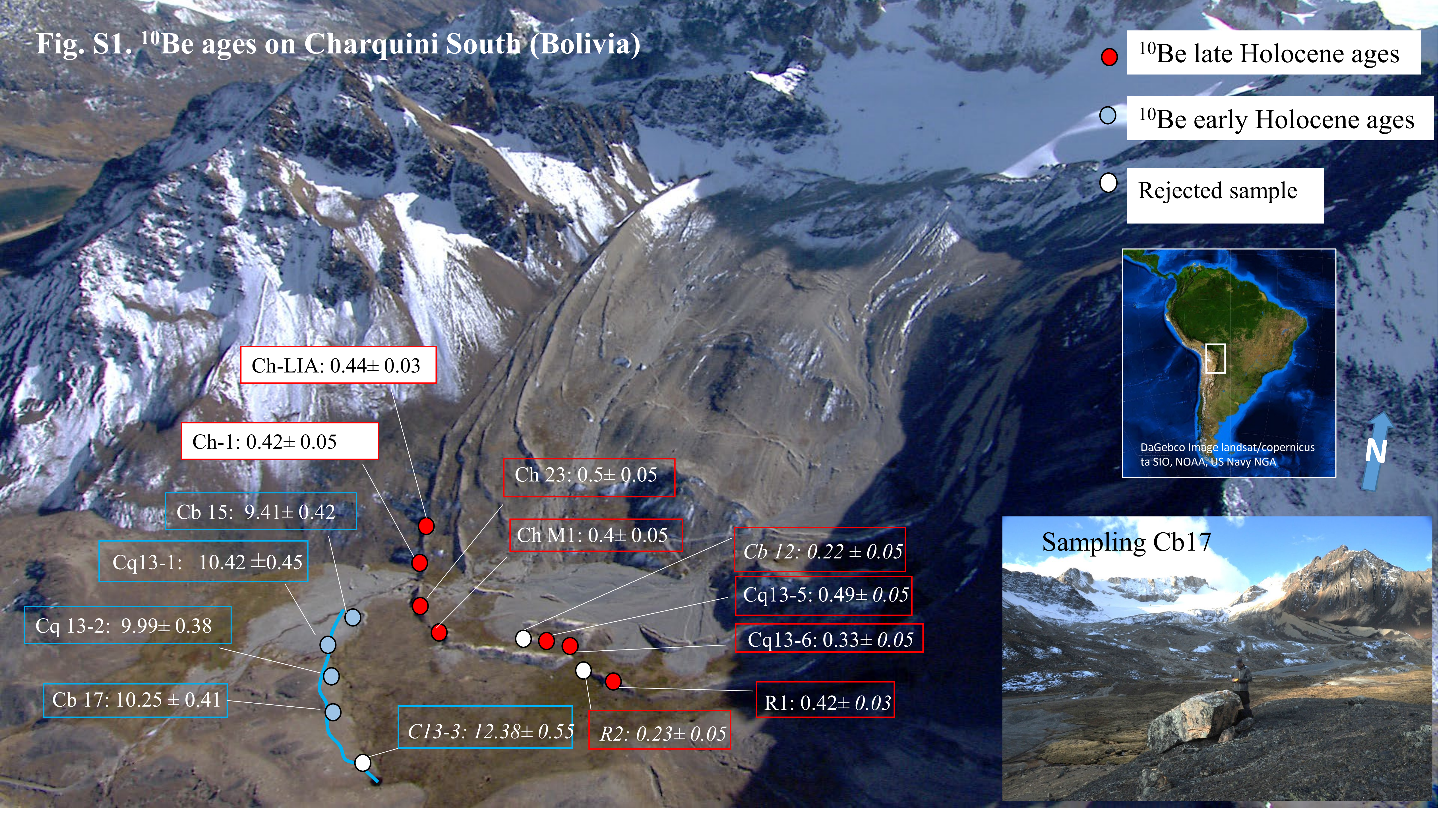

Fig. S1. ^{10}Be ages on Charquini South (Bolivia)
^{10}Be late Holocene ages
^{10}Be early Holocene ages
Rejected sample
DaGebco Image landsat/copernicus
ta SIO, NOAA, US Navy NGA
N
Sampling Cb17
Ch-LIA: 0.44± 0.03
Ch-1: 0.42± 0.05
Ch 23: 0.5± 0.05
Ch M1: 0.4± 0.05
Cb 15: 9.41± 0.42
Cq13-1: 10.42 ±0.45
Cq 13-2: 9.99± 0.38
Cb 17: 10.25 ± 0.41
C13-3: 12.38± 0.55
Cb 12: 0.22 ± 0.05
Cq13-5: 0.49± 0.05
Cq13-6: 0.33± 0.05
R1: 0.42± 0.03
R2: 0.23± 0.05

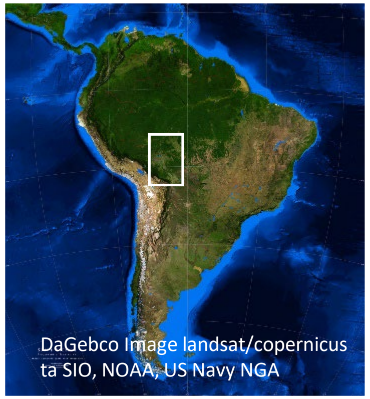

Moraine samples

- ^{10}Be late Holocene ages
- ^{10}Be early Holocene ages
- Rejected sample

Bedrock samples (roche moutonnée)

- Upper sample area
- Lower sample area

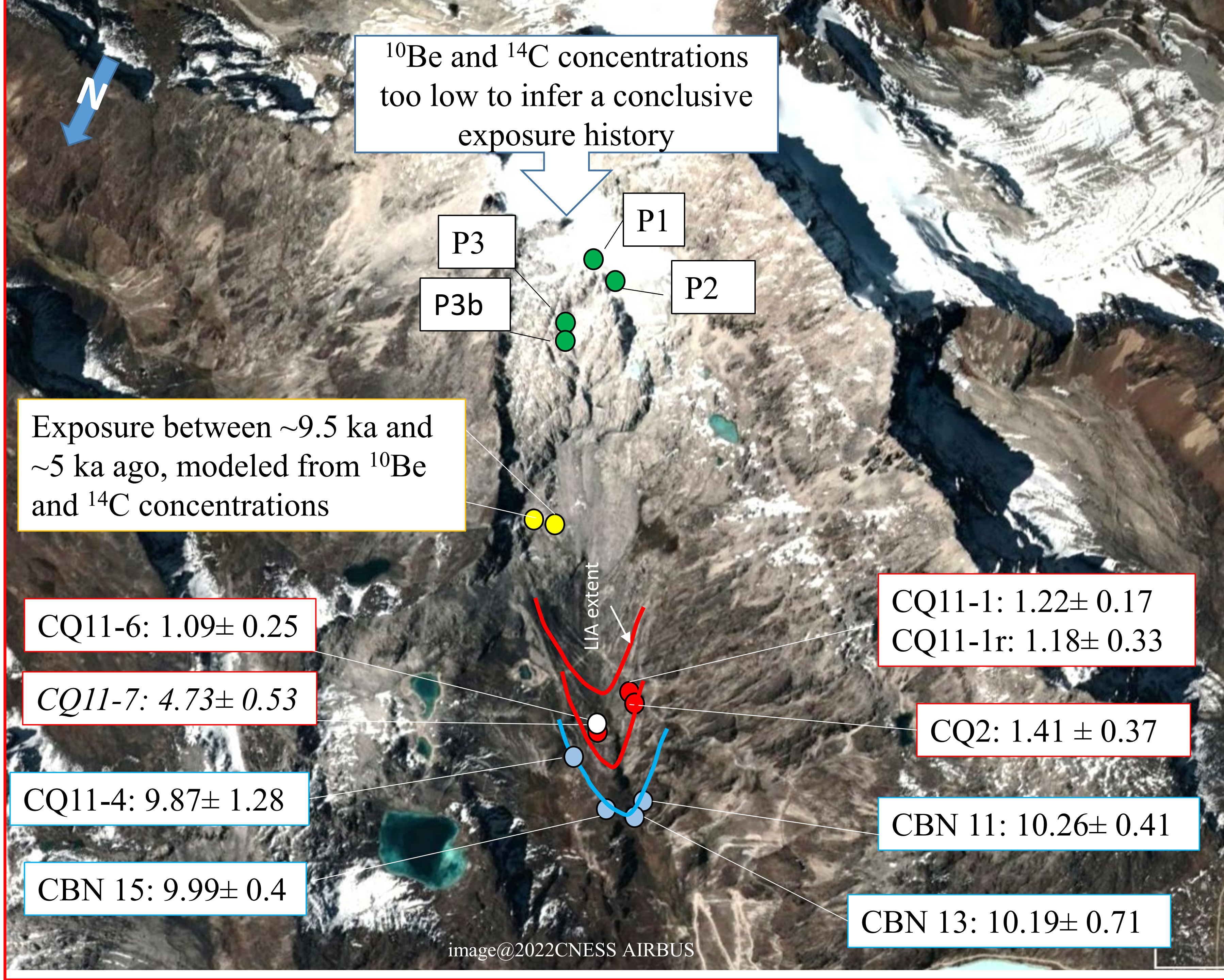

Fig. S2. Moraine and bedrock sample locations at Charquini North (Bolivia)

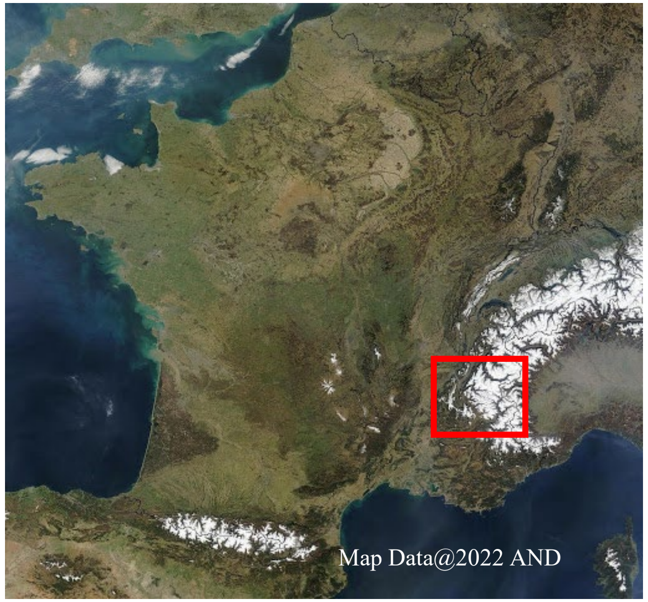

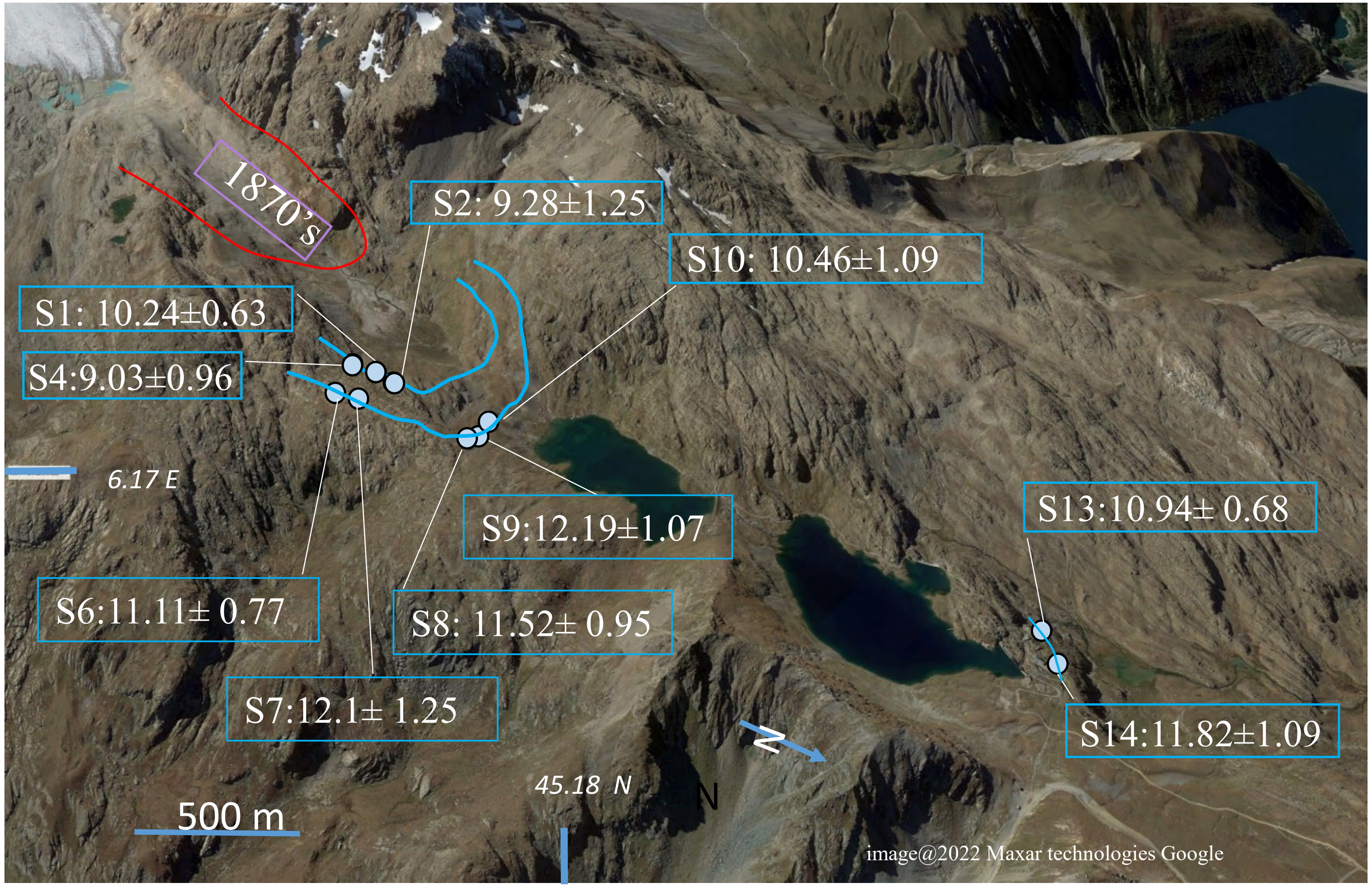

Fig. S3. ^{10}Be ages on St Sorlin glacier (French Alps)

^{10}Be early Holocene ages

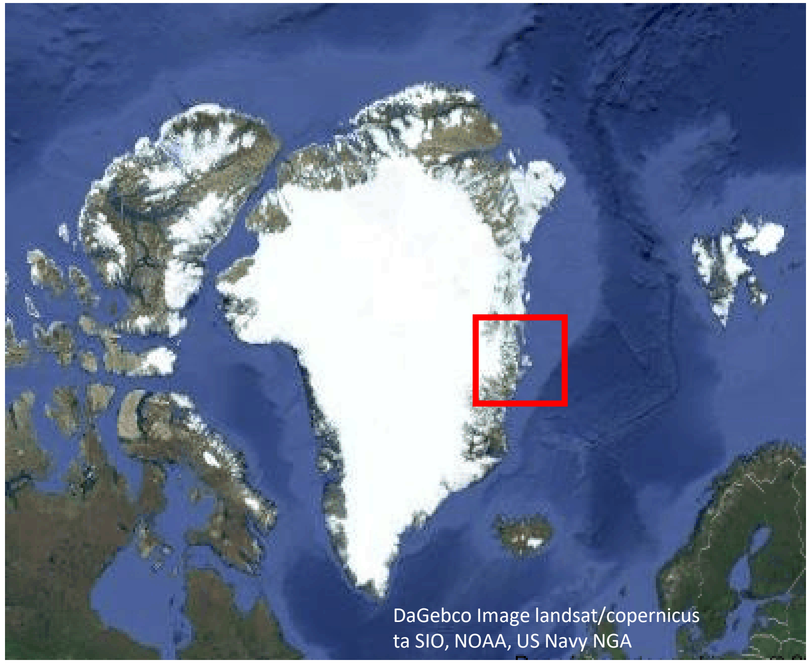

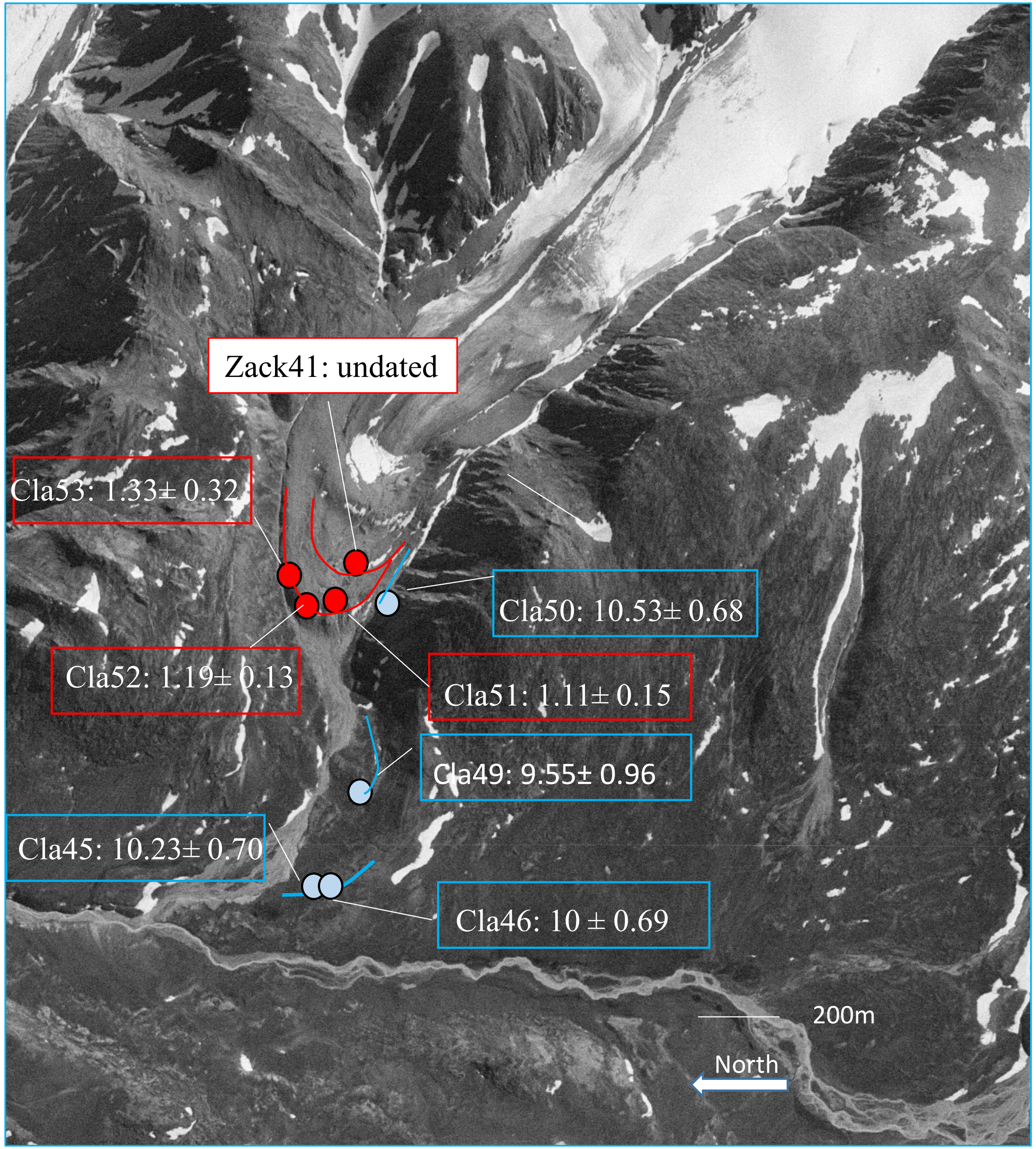

● ^{10}Be late Holocene ages

● ^{10}Be early Holocene ages

Fig. S4. ^{10}Be ages at Clavering (East Greenland)

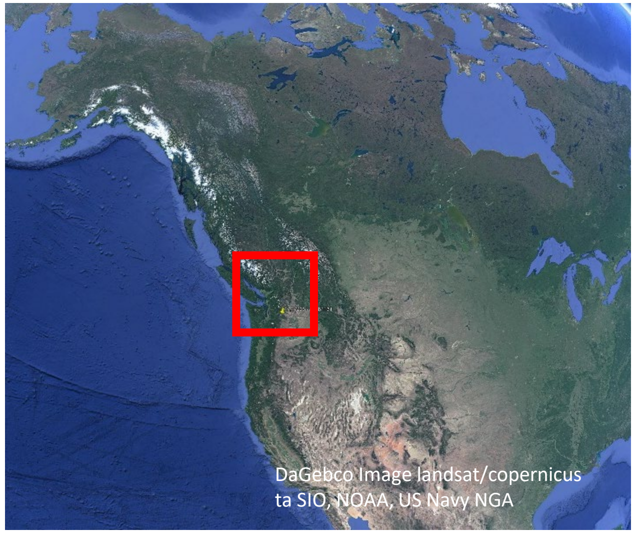

● ^{10}Be late Holocene ages

Early Holocene moraine after Marcott et al. (2019).

Fig. S5. ^{10}Be ages at Enchantment Lakes (Cascade Range, US)

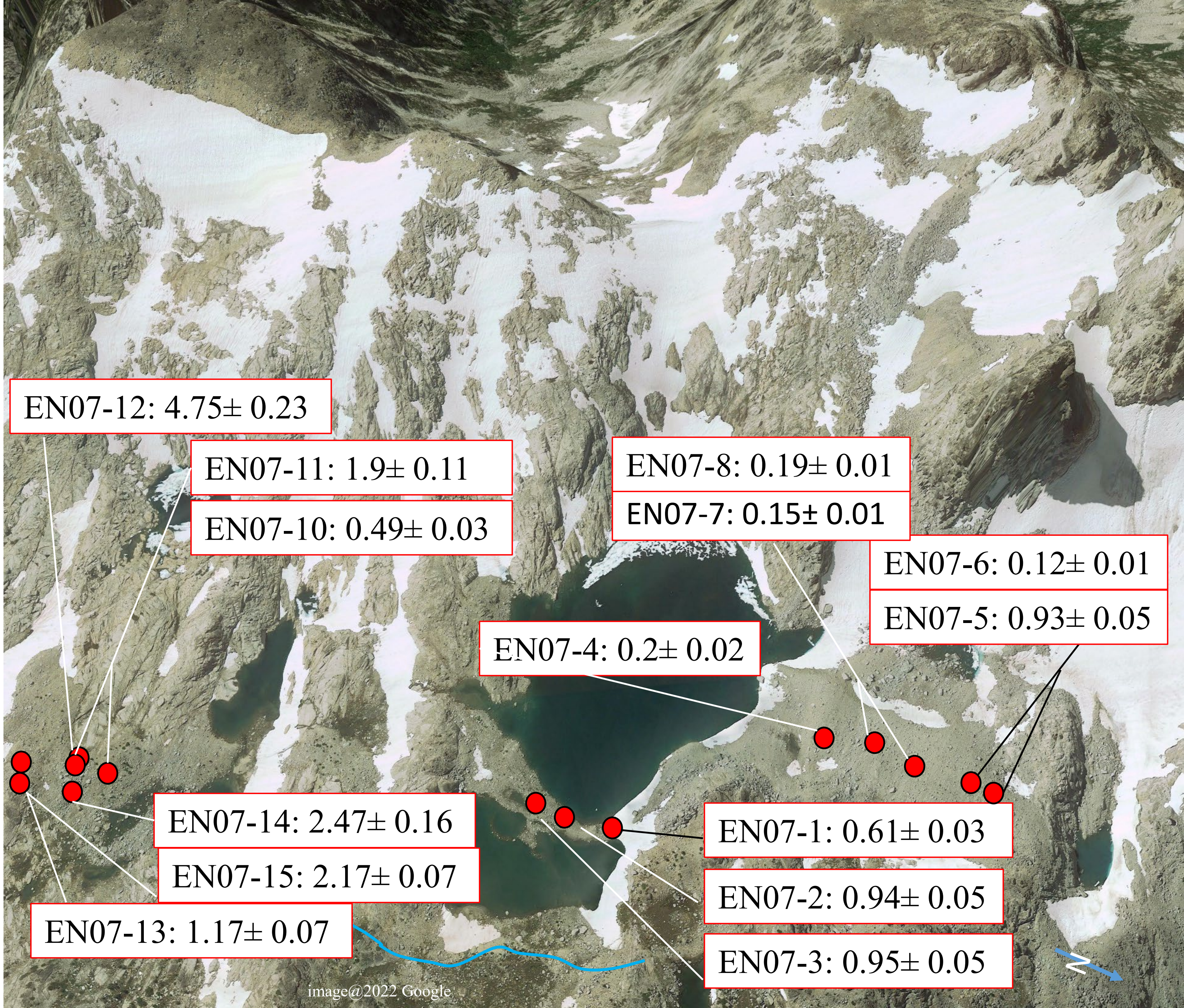

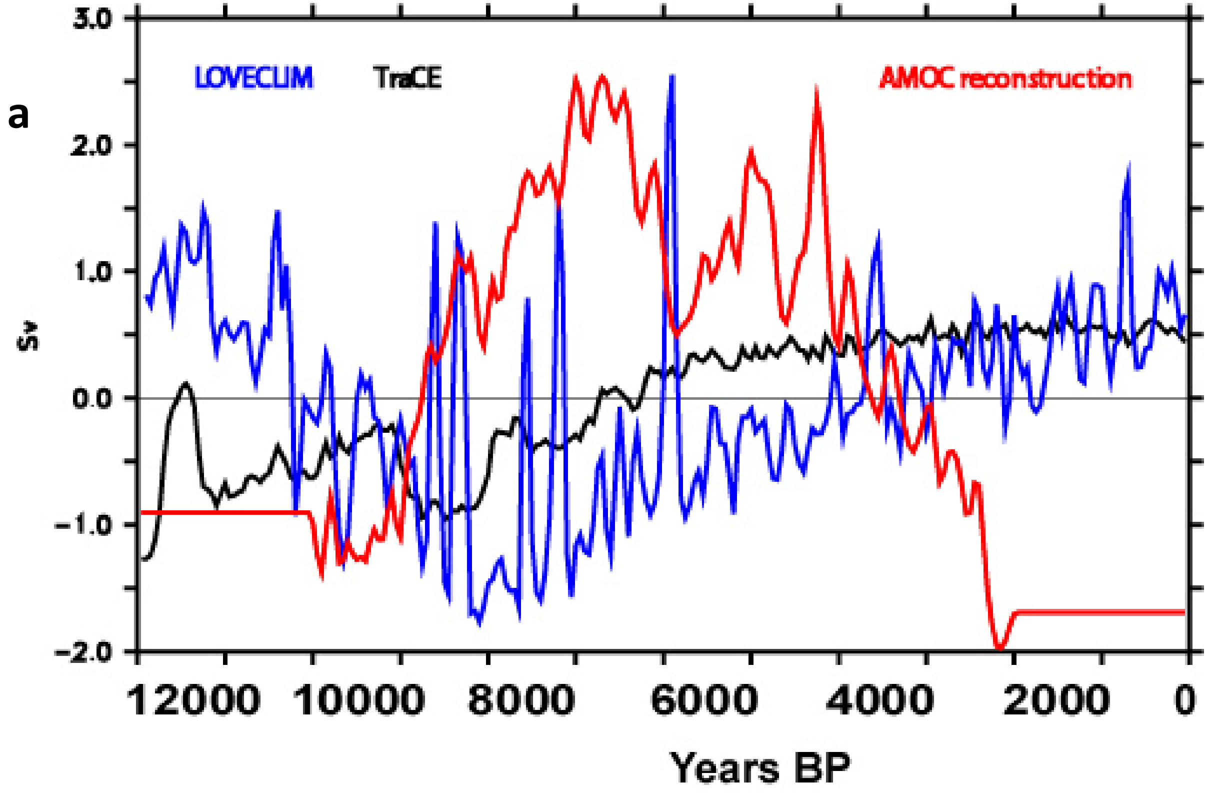

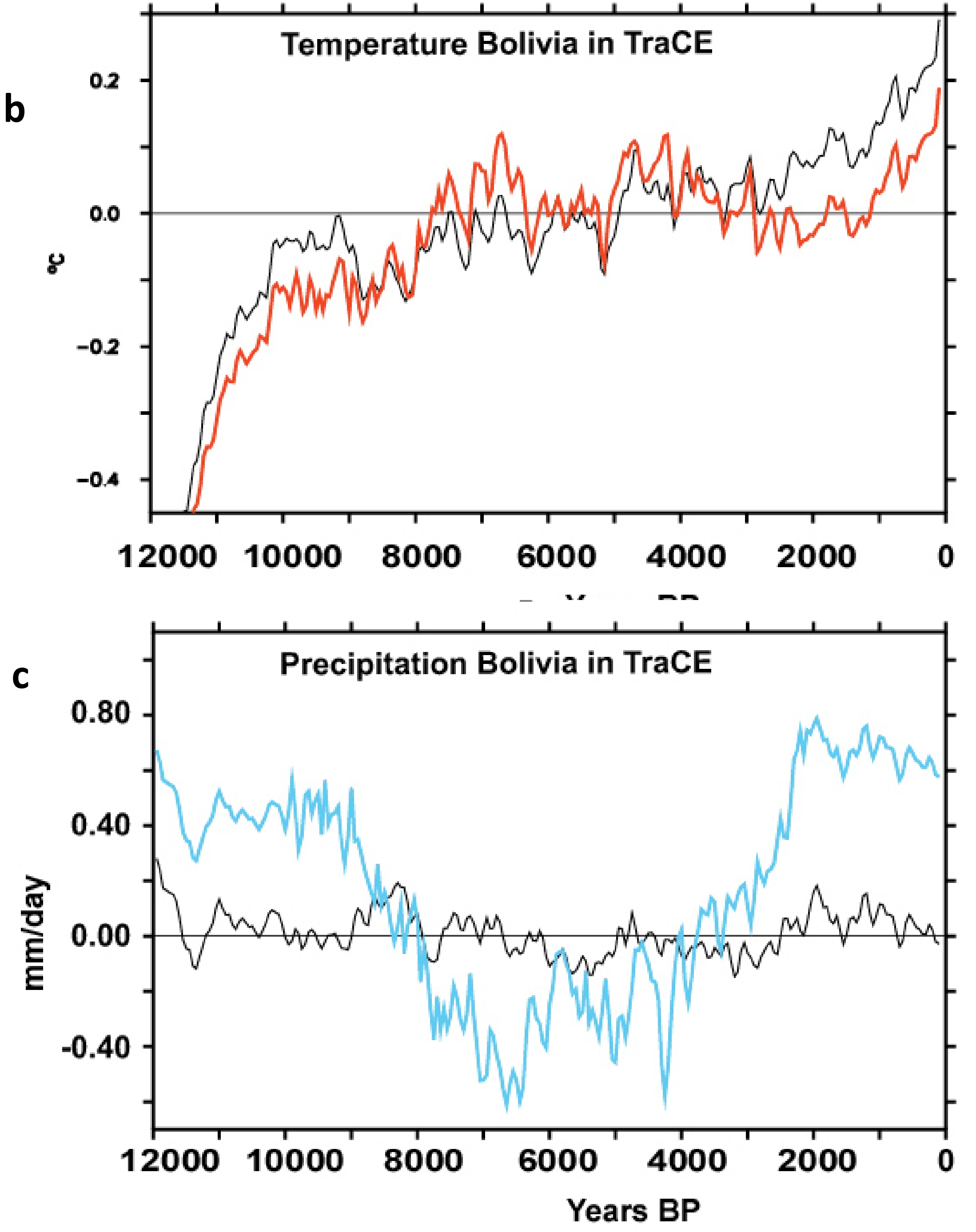

Fig. S6. Holocene AMOC changes from models and reconstructions and its influences on temperature and precipitation changes in Bolivia. **a** AMOC indices at 26°N (in Sv). Reconstruction calibrated over the Holocene (see Methods) in thick red, in the LOVECLIM transient simulation in blue and in the TraCE similation in black. A 100-yr running mean has been applied to the transient simulation to easy readability. **b** temperature and **c** precipitation in Bolivia from TraCe with raw data in black and AMOC corrected data in orange and magenta.

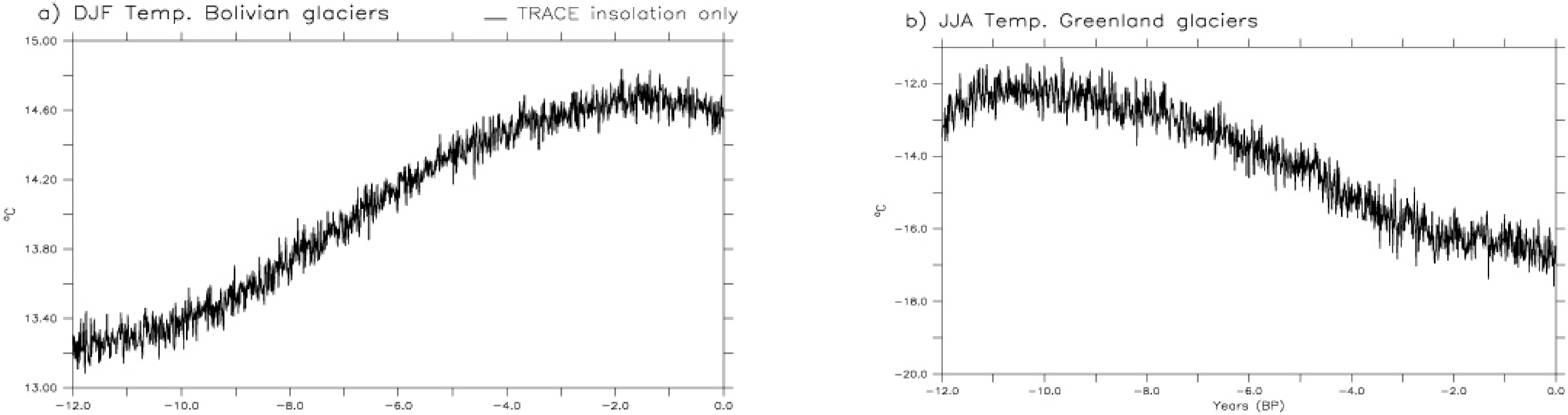

Fig. S7. Holocene summer temperature changes estimated from TraCE experiment. a) Bolivian region (Long 290°, Lat 16°); b) Northeast Greenland (Long 339°, Lat 74°)

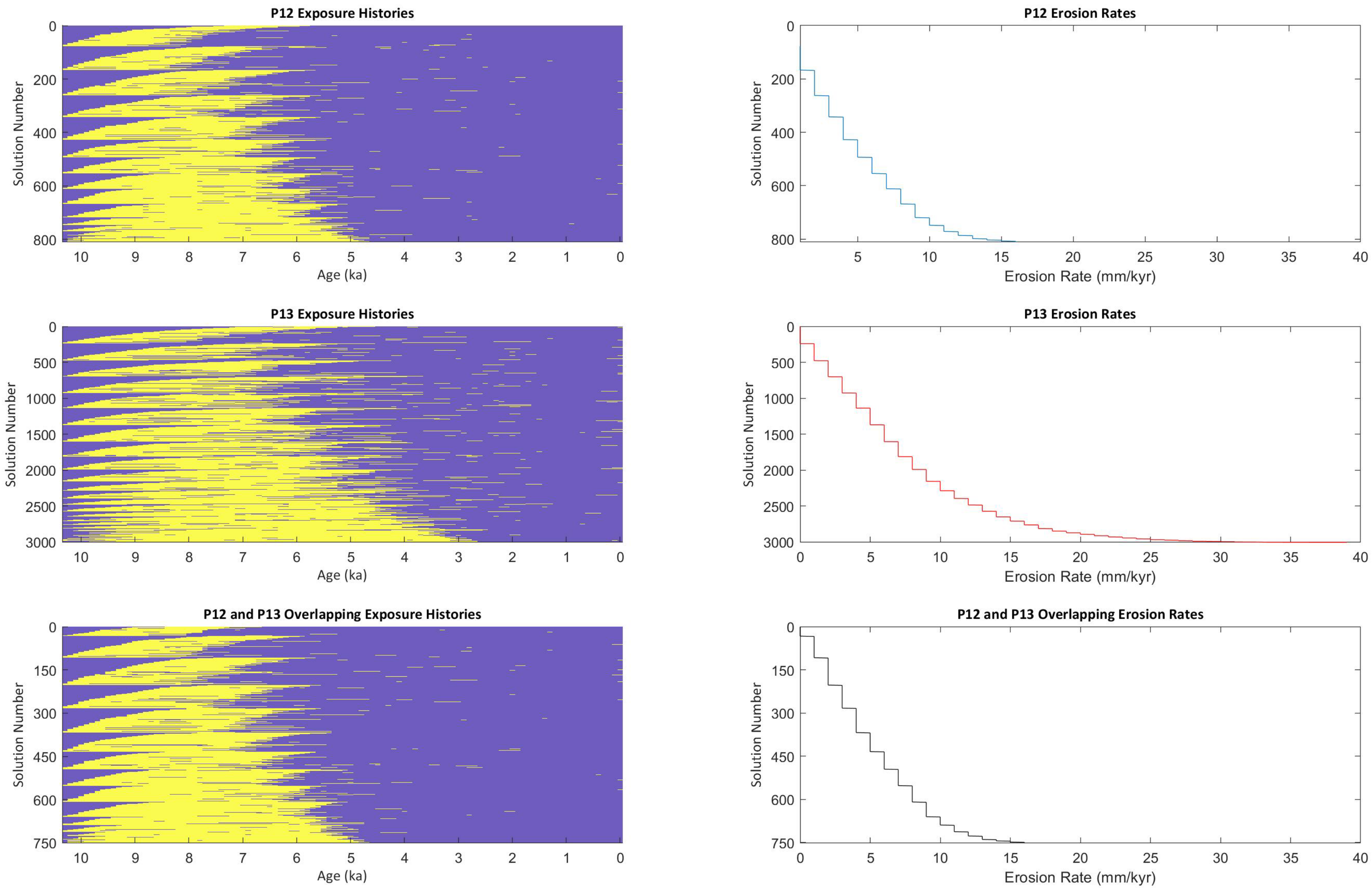

Fig. S8. Exposure histories (left) with yellow indicating exposure and purple indicating burial, and corresponding subglacial erosion rates (right) that yield ^{10}Be and ^{14}C concentrations within 3σ of measured values of Charquini North (Bolivia) bedrock samples. The top two panels show results for samples P12 and P13 individually, and the bottom panel shows results consistent with both P12 and P13.
