# Supplementary material for: In-phase millennial-scale glacier changes in the tropics and North Atlantic regions during the Holocene
Source: Nat Commun. 2022 Mar 17;13:1419. doi: 10.1038/s41467-022-28939-9 (PMC8930989; doi:10.1038/s41467-022-28939-9)
Supplement: Supplementary file 3 — Description of Additional Supplementary Files [file 41467_2022_28939_MOESM3_ESM.pdf]

Description of Additional Supplementary Files

File Name: Supplementary Data 1
Description: Cosmogenic 10Be exposure ages and in situ 14C data

File Name: Supplementary Data 2
Description: Glacial extent used in Figure 1. Data are collected from publication and are grouped by region/zone.
